# Musical Hallucinosis: Auditory Illusions After Hearing Loss and Cochlear Implantation

**DOI:** 10.1177/15459683251348171

**Published:** 2025-06-19

**Authors:** Bruce H. Dobkin

**Affiliations:** 1 Department of Neurology, Geffen/UCLA School of Medicine, Los Angeles, CA, USA

**Keywords:** hearing loss, cochlear implant, tinnitus, musical hallucinations, auditory illusions, auditory neuroplasticity, sensory rehabilitation, Bayesian inference

## Abstract

**Background:**

Phantom auditory percepts are especially prevalent in healthy persons with hearing loss. No first-person description of the not uncommon illusion called musical hallucinosis (MH) has been published in relation to possible neural mechanisms for its occurrence.

**Objectives:**

The author presents his personal experience following implantation of a unilateral cochlear neuroprosthesis to try to compensate for progressive sensorineural hearing loss.

**Results:**

The MH included abrupt onset of persistent, robust singing of the Star-Spangled Banner, then other familiar songs and nursery rhymes by a men’s choir without accompanying instrumentals, followed months later by continuous nonsense lyrics sung to a simpler stereotyped tune. The onset was associated with deafness as a complication of electrode placement within the cochlea, the early sizzling, synthetic, monotonal auditory sounds heard using the cochlear implant, and a burst of cacophonous tinnitus following a higher volume adjustment to the device.

**Conclusions:**

Several physiological alterations, including deafferentation-induced spontaneous auditory pathway activity that triggers higher auditory cortical areas to place the ambiguous inputs within the individual’s prior experience of sound patterns, may help explain the evolution of MH and its persistence as a type of maladaptive neuroplasticity.

## Introduction

We are all explorers who subconsciously use prior experience and context to make sense of entangled patches of ambient sounds and speech. Momentarily thinking you heard someone call your name in a crowd is a common experience. In surveys, 15% of healthy persons with normal hearing acuity have gone a step farther, occasionally hearing a disembodied voice.^1^ With hearing loss, episodic auditory hallucinations other than tinnitus may have a prevalence as high as 16% in adults, but unlike people with psychotic hallucinations, they have good insight and no delusions, paranoia, or thought disorders. Individual hearing specialists report this complaint, however, only about once every 6 months, which suggests that patients are not routinely being queried or forthcoming about such phenomena.^2^

Musical hallucinosis (MH) is perhaps the most peculiar repetitive auditory intrusion. I describe my own experience in relation to case reports about MH and consider several theoretical mechanisms.

## The Experience of MH

I had progressive age-related hearing loss for 25 years. My binaural acuity became so poor that I had to gradually withdraw from social interactions, then lecturing, research, and patient care, finally retiring from a career as an academic neurologist. I reached out for a right-sided cochlear implant (CI), aiming to regain enough speech discrimination through auditory rehabilitation to better enable daily communication.

My CI includes a magnetic receiver implanted under the scalp that connects to the 22 wired electrode frequency channels burrowed tonotopically along the spiral ganglia of the cochlea and its auditory nerve. The microphone, sound processor, and battery rests over the upper pinna, wired to a magnetic scalp transmitter. When the magnets are placed in contact, the CI activates. A month after the first contact, an audiologist increased the loudness of processor inputs, one of many typical adjustments. The next evening, crashing metal pots and pans, clashing cymbals, and high-pitched clanging bells startled me awake. I searched out the source, checking the upstairs rooms, opening front and back windows as the sun rose, then looking downstairs. The cacophony did not vary and persisted whether or not the CI was connected. After 6 nights of poor sleep under this continuous, assaultive tinnitus-like hallucination, deep silence greeted me. The phantom, thunderous reverberations never returned. Nor did the far less disturbing right-sided ringing tinnitus that had accompanied my progressive deafness.

The next morning, however, softly and then more convincingly, I heard a men’s choir of tenors and baritones belt out the *Star-Spangled Banner*. I searched for the singers. Their voices persisted everywhere, whether or not the speech processor’s transmitter was clipped to my scalp. The patriotic national anthem seemed off to my right, a loop replaying every 62 seconds, non-stop. About 50 dB of white noise from a smartphone app and directed into the CI microphone, equal to the loudness of a group conversation, masked their song. I had never before hallucinated singing or voices and was not prone to obsessive thoughts. The robust singing, unaccompanied by instruments, persisted for 3-1/2 weeks, day and night, then suddenly ceased.

Within hours of the last sung stanza, new tunes and words took the place of the anthem. The vocalists belted out the first verses of nursery rhymes and childhood songs—Yankee Doodle, Old McDonald, Mary and her lamb, Go Down Moses, and a dozen others. The tunes were just as I had learned them as a child, but shifted from one to another within a few minutes. No more recent melodies played. The choir sung with gusto and lyrical clarity every waking hour and interrupted light sleep overnight. The medley was nothing like a stuck song or so-called earworm, as in a familiar catchy tune with a portion of lyrics that replay in one’s head. I could not alter the tune or words or mentally block it out. During a momentary break in my conversations, a background ditty filled the silence until the conversation restarted.

It was still early in my long rehabilitation process of relearning to recognize CI input when the phantom chorale invaded. Spoken words were still monotonic and synthesized, and I did not yet recognize the voices of my wife and children. Speech, environmental sounds, and music also sizzled like syllables frying in bacon grease, which seemed far more alien than the choir’s natural sounding lyrical output.

About 8 months later, the choir gradually turned the pages of its hymnal to include robustly sung nonsense lyrics in endless loops of mostly the same 4 to 6 notes per bar in a repetitive rhythm. By a year after the onset of MH, familiar songs ceased and the nonsense lyrics prevailed. The baritones faded away, leaving only lusty tenors. Each stereotyped tune persisted a few minutes with variations in loudness and word clarity, then changed to words that had no apparent connection to prior ones. I could not manipulate the lyrics or the tune with my thoughts or by listening to other music. Hundreds of different exuberant repeating stanzas have haunted me, such as:

Oh change a lot, oh change a lot

Oh change, oh change, oh change a lot.

Shark shark, shark shark, shark shark away

Shark shark, shark shark, shark shark awa-ay.

Fifty one oh one, fifty one oh one

Fifty one oh one, fifty one oh one.

Pull-ee, pull-ee, pull-ee pull-ee

Pull-ee, pull-ee, pull-ee, pull-eeee.

Won ton day, itsa won ton day

Itsa won ton day, itsa won ton day.

I had been hacked by a spectral choir. I trained myself to mostly ignore this elephant in my mind in fear that I might reinforce the hold of the tune and lyrics in my brain. That helps—by a few minutes after one nonsense ditty segues to another, I usually have no recall of prior content. When trying to fall asleep or if awakened during the night, I try to repel my attention to the choir with meditative breathing, which had been my tactic for bedtime tinnitus—slow inhale, hold a few seconds, then slow exhale while concentrating on the feeling of air flow, until asleep. That is one of the methods of auditory rehabilitation for tinnitus and MH, which emphasize cognitive behavioral therapy, education about the phenomenon, and counseling to assist with coping.

I experimented for a few months with trials of medications suggested by case reports in which the loudness of the MH sometimes decreased.^3^ My trials failed to soften or unplug the choir’s jukebox. I considered types of inhibitory noninvasive brain stimulation alluded to for those without a CI, but that energy might heat up or demagnetize the implanted skull receiver.

### Other Reported Experiences

MH has occasionally been reported in association with stroke, subcortical white matter lesions, brain tumors, epilepsy, and neurodegenerative disorders.^4^ Sensorineural hearing loss is a far more common precursor. A semi-structured interview of 194 patients referred for audiology assessment who had mild to moderate hearing impairment found that 4% described MH.^5^ No particular risk factors were found, including age, severity of hearing loss, or presence of tinnitus. Only a few had ever mentioned it to their doctors, despite the persistence of MH for up to a decade. Perhaps the percept was not bothersome or they did not want to face the potential stigma often associated with hallucinations.

Can MH be induced by the new input from a CI? In retrospective, cross-sectional surveys, 28% of 350 CI recipients^6^ and 22% of 82 others^7^ described MH, but half had it before implantation. Over 50% had never informed a health professional. A minority had continuous MH. About 75% of those with new MH heard it on the side of the first implanted CI. So novel CI input may contribute. On the other hand, just as hearing amplification with aids may suppress tinnitus, occasional reports reveal that a new CI may suppress chronic MH. Another finding in those surveys was that 10% of 1 group^6^ and the majority of the other^7^ felt their MH had an adverse emotional effect or was intolerable, but 40%^6^ found it pleasant.

First-person descriptions of MH that include details about the experience are infrequent. Most brief accounts mention, as in my case, that the music is often familiar, includes childhood ditties, and usually takes the form of stirring religious, patriotic, or drunken pub songs that can vary in content and duration, lasting for minutes, hours, days, or months. In 3 of his books and a commentary,^8^ Oliver Sacks mentioned a range of MH experiences from several patients and from hundreds of letters sent to him from the community. Most occurred in association with sensorineural hearing loss. Many in this self-selected group had continuous hallucinosis. Bing Crosby singing *White Christmas*, for example, might begin abruptly as if on a radio in another room. The recipient could not turn it off or change the volume. Unlike me, some could alter the lyrics, speed, or harmony of songs. Sometimes the phantom singers or instrumentals were loud enough to interfere with listening during conversations. That has never happened to me. Many also experienced familiar melodies or instrumentals from their own playing repertoire. After the death of a spouse, a mourner heard languid instrumental music, loud and clear, but unfamiliar. I have never heard instruments, only my men’s choir, which pays no attention to my mood.

Two years after onset, my MH continues with newly conjured, nonsensical lyrics to a minimally altered tune, masked only by ambient sound that exceeds 35 dB when I am wearing the CI, which I cannot overnight. White noise at 80 dB heard by my left ear without an aid can hide the MH, but of course prevents sleeping. The choir has been a vexing nuisance in that it has stolen silence and interrupted sleep. Although the CI has restored my hearing of pure tones and speech in a quiet environment to 65% of normal, the illusions have made me reluctant to get a left-sided CI to further improve binaural hearing. My fascination with its neural underpinnings, however, has been a refuge. MH may share some of the neural computations that help explain normal auditory illusions, tinnitus, and non-auditory sensory misperceptions.

## Auditory Illusions

Auditory illusions, in the absence of psychosis, have been reported in multicultural surveys at prevalence rates ranging from 4% to 20%.^1^ They were frequent and persistent, however, in only 4% of the voice hearers. The normal visual phenomenon of seeing, for example, cartoon faces in inanimate objects, called pareidolia, has been extended to hearing indistinct voices or music in the random noise of, for example, a running fan. Central mechanisms, perhaps related to genetics or epigenetics (eg, particular neurotransmitter levels), have been suggested as predisposing some people to be more prone to auditory, visual, and other sensory illusions.^9,10^

Hearing loss, despite the use of hearing aid amplification, can cause acoustical inputs to become less precise and more ambiguous, associated with cochlear, brainstem, and auditory plus associational cortex physiological and structural plasticity.^11^ This adaptive and maladaptive plasticity may contribute to illusions. In parallel, reduced visual acuity and blind spots due to glaucoma, macular degeneration, or partial visual field loss create visual ambiguities and the visual hallucinations of the Charles Bonnet syndrome.^12,13^

Listening with my CI, running motors still embed the auditory illusion of a distant rapid conversation as if between 2 podcast hosts speaking over each other. The flow of water from a faucet or down the shower and toilet drains also carries a conversant mix of pitches akin to very rapid chatter. While tinkling into a toilet bowl, similar rapid staccato chatter sometimes bubbles up a word, such as *justice* or *good evening*, which is most amusing. Higher cortical regions seem to be attempting to make sense of the still somewhat ambiguous auditory input from the CI, just as hearing loss alone might.

In experiments, the brain has often been shown to fill in missing or degraded signals from sensory inputs to work around mismatches between predicted and received information.^14,15^ Meaningful perceptions arise from the optimal integration of prior expectations built from past experience about similar inputs in relation to the incoming sensory evidence that may be noisy and ambiguous, especially due to hearing loss. If prior expectations are more precise, that is, less uncertain than the sensory evidence from degraded auditory input, then hallucinatory perceptions of tinnitus, voices, instrumentals, singing, and other sounds may emerge. Models of the common illusion of tinnitus often include the impact of spontaneous, disinhibited brainstem auditory neuron activity that follows inner and outer cochlear hair cell loss.^16-19^ This synaptic chatter ascends through the hierarchy of cortical auditory regions, in a sense seeking conscious meaning. That spontaneous neural noise may also contribute to MH in those with progressive or sudden hearing loss.

## Physiologic Contributions to MH

Based on available experimental literature and my experience, how might my MH have evolved? At least 4 interactive sources may have contributed. First, I had lived with the effects of disinhibited brainstem auditory neuronal activity that presumably caused my ringing tinnitus for decades, so this maladaptation was well established. The sudden, complete deafferentation-induced deafness during CI surgery may have initiated even greater disinhibition of that neural noise. With this new baseline, the upward adjustment in the power of the CI’s channels 4 weeks after it was first turned on immediately kicked off very loud tinnitus-like noise, perhaps by further amplifying that spontaneous auditory brainstem neural activity. In addition, the CI at that time transmitted louder, but rather low signal-to-noise sound to the brainstem and higher cortical regions, which had only begun to engage the practice-induced neuroplasticity necessary for later gains in word sound discrimination. These still ambiguous brainstem and lower cortical auditory inputs may have led to a loop of recurrent excitation and inhibition within music-associated higher cortical regions, subsequently settling on a percept without a stimulus,^20^ my choir.

More specifically, Kumar et al^20^ described a model that considered the brain as a Bayesian inference prediction machine that aims to make sense of nonsense. Based upon a magnetoencephalography study in 1 subject with MH, they suggested that the anterior superior temporal gyrus (a site involved in melody) may sit at the top of a hierarchical loop for sound interpretation. This area was hypothesized to convey relatively precise auditory prediction errors about ambiguous internal auditory noise to the motor area (site for musical imagery) and the posteromedial cortex, a site for retrieval of musical melody from memory that also generates musical imagery. The latter 2 regions were thought to reciprocate neuromodulating predictions and error corrections to the anterior superior temporal gyrus. These 3 regions, then, were engaged in a form of self-organized, reinforcement learning. Their recurrent loop of communication subsequently no longer had to be entrained by the imprecise bottom-up brainstem and primary auditory cortex inputs, having crystalized into the autonomous perceptual network for MH.

## Tunes and Words in MH

Why music? Music, compared to speech, may offer a more predictable auditory percept for the brain.^20,21^ Musical sounds, with their discrete pitches, volumes, and tempo in relation to each other have statistical properties or rules such that several notes readily predict the next note in a musical bar. Music, then, is usually more predictable than speech and better proceeds within expectations. Melody and lyrics are also often repetitive and may evoke emotions and memories, further strengthening their reinforcement. Thus, a weak, ambiguous auditory percept may arise and strengthen within the powerful properties of music, leading to autonomous instrumentals, songs, or both that exist beyond conscious control. Of note, neuroimaging and neurophysiological studies reveal that MH activates, at minimum, the same regions as would any external source of music.^21-23^ Many of these regions are also active in tinnitus.^24^

The lyrics and tunes of MH may primarily arise from the nearby parahippocampus,^20^ given its role in encoding auditory input for memory and the finding that the region is always activated in neuroimaging studies of MH. This region, of note, is also active during hallucinations in schizophrenia. In those with MH, the uncertainty created by ambiguous sound inputs facilitates the region to try to fill in missing auditory information from memory. While that may help explain the frequency of memorable tunes from childhood, along with religious and patriotic songs, it does not quite capture the meaningless words that some of us experience. In my case, the individual word sounds are always a bit ambiguous and comprised of only 1 to 2 patterns of syllables, perhaps making for flexible substitutions.

## Conclusion

As my experience and reports suggest, the content and psychological burden of MH will differ across individuals and over time, perhaps related to variations in auditory experience prior to and with the ongoing MH, a genetic predisposition to sensory illusions, and perhaps differences in the circuitry involved.

Better medical recognition of the presence of MH in hearing-impaired persons and in post-lingual persons who receive a CI, perhaps obtained simply by routinely asking about such symptoms, would enable clinicians to better appreciate and manage their effects on health-related quality of life. Identification of those affected could also allow researchers to identify participants for longitudinal, mechanistic, and interventional studies. The bases for phantom auditory perception and MH also have much to offer for studies of neuroplasticity and about how we come to make sense of our auditory landscape.
